# Frequency and risk factors of psychological distress among individuals with epilepsy before and during the outbreak of the SARS-CoV-2 Omicron variant in China: an online questionnaire survey

**DOI:** 10.1186/s42494-023-00146-z

**Published:** 2024-01-15

**Authors:** Xiaoting Hao, Qi Zhang, Chenxi Zhong, Enzhi Li, Yingqi Jiang, Jiajun Xu, Yuanyuan Li, Dong Zhou, Bo Yan

**Affiliations:** 1 Department of Neurology, West China Hospital, Sichuan University No, 37 Guoxue Alley, Chengdu, Sichuan Province 610000 China; 2 Department of Psychiatry and Mental Health, West China Hospital, Sichuan University/West China School of Clinical Medicine, Sichuan University, Chengdu, 610000 China; 3 Department of Neurology, Chengdu Shangjin Nanfu Hospital, Chengdu, 611730 China; 4 West China School of Nursing Sichuan University, Chengdu, 610000 China; 5 Department of Clinical Research Management, West China Hospital, Sichuan University, Chengdu, 610000 China

**Keywords:** Epilepsy, Neurological symptoms, Psychological distress, COVID-19 pandemic

## Abstract

**Background:**

The COVID-19 pandemic substantially increases the risk of severe psychological distress among people with epilepsy (PWE), especially those with monthly household income < 5000 RMB or with uncontrolled seizures. Patients with Kessler scores > 12 should consult a psychiatrist, especially during major disasters. This study was aimed to compare the frequency of psychological distress among Chinese PWE before and during the outbreak of the SARS-CoV-2 Omicron variant, and to identify risk factors for such distress.

**Methods:**

In this prospective study, we collected sociodemographic data of PWE aged > 14 years, who were treated at our center during December 1 to 15, 2022. All participants completed the 6-item Kessler Psychological Distress Scale before the outbreak and again during the outbreak. Health visitors who were unrelated to those patients during the outbreak were included as a control. Multivariate logistic regression analysis was performed to identify risk factors of severe psychological distress and its exacerbation.

**Results:**

Of the 223 PWE, 127 were tested positive for SARS-CoV-2, while 174 of 218 controls were positive for SARS-CoV-2. The neurological symptoms were similar between PWE and controls with SARS-CoV-2. The average Kessler score of PWE was significantly higher during the outbreak than before it (9.93 ± 3.98 vs. 8.52 ± 0.23, *P* < 0.001). The average score of controls during the outbreak (5.146 ± 0.35, *P* < 0.001) was significantly lower than that of the PWE. We identified three independent predictors for severe psychological distress in PWE during the outbreak, i.e., monthly household income < 5000 RMB (OR = 0.252, 95%CI 0.064–0.998,* P* = 0.048), severe psychological distress before the outbreak (OR = 0.067, 95%CI 0.026–0.174,* P* < 0.001), and seizure onset within 30 days before the assessment during the outbreak (OR = 0.356, 95%CI 0.157–0.805, *P* = 0.013). Of the three predictors, the last one was also an independent predictor for exacerbation of psychological distress during the outbreak (OR = 0.302, 95%CI 0.123–0.741, *P* = 0.009).

**Conclusions:**

Our analysis suggests that the COVID-19 pandemic has substantially increased psychological distress of individuals with epilepsy, regardless of the viral infection or not. Various neurological symptoms similar to those of “long COVID” appeared for the first time among these individuals during the Omicron outbreak, highlighting the need for clinicians to screen carefully for this condition. Management of epilepsy during the pandemic or a similar major disaster should focus on the control of seizures and maintenance of mental health, especially among those with monthly household incomes below 5000 RMB, suffering uncontrolled seizures and having a history of severe psychological distress.

**Supplementary Information:**

The online version contains supplementary material available at 10.1186/s42494-023-00146-z.

## Background

Epilepsy is a chronic neurological disease that affects individuals of all ages, with a global lifetime prevalence of 7.6 per 10,000 [1]. Epilepsy poses a heavy health and economic burden on affected individuals and the society, especially since about half of adults with active epilepsy have at least one comorbidity [2, 3]. For example, people with epilepsy are eight times more likely to have a mental disorder [4, 5] and three times more likely to commit suicide [6] than the general population. As the most frequent comorbidity of epilepsy, mental illness shortens the life expectancy, reduces the quality of life [7], lowers medication compliance and worsens the side effects of antiseizure medication. Early recognition and treatment of psychiatric comorbidities is critical for improving the prognosis of individuals with epilepsy [8].

During the past three years of COVID-19 pandemic, more than 650 million cases and 6.5 million deaths have been documented, and the number of affected individuals with long-term sequelae has been growing [9]. Even when infection with the causative SARS-CoV-2 virus is asymptomatic or mild, individuals may still develop neurological and psychiatric symptoms, including sleep problems, anxiety, and depression [10]. This potential of negative effects on mental health is especially great in China following the introduction of adjusted COVID-19 prevention and control measures in December, 2022 [11]. Infection with SARS-CoV-2 is not the only risk factor for mental problems related to COVID-19: the pandemic can also exert substantial indirect effects on uninfected people [12]. For example, we have found that the COVID-19 pandemic has increased the risk of severe psychological distress among uninfected individuals with epilepsy [8].

In this study, we compared the frequency and intensity of psychological distress in individuals with epilepsy before and during the outbreak of the SARS-CoV-2 “omicron” variant in China. We also explored risk factors for severe psychological distress and exacerbation of psychological distress during the pandemic.

## Methods

### Participants

This prospective case-control study was conducted from December 1, 2022 to February 15, 2023, Patient information was prospectively entered into a database that has been described elsewhere [8]. Due to the sudden outbreak, it was impossible to recruit the control participants before adjustment of the COVID-19 prevention and control measures. Therefore, the control group comprised health visitors unrelated to those patients recruited during January 15 to February 15, 2023. This study was approved by the Ethics Committee of Sichuan University, and participants gave informed consent before enrollment.

Individuals with epilepsy who were diagnosed at the Epilepsy Center of West China Hospital (Chengdu, China) between August 2015 and December 2021 were invited to participate. They were at least treated for 1 year before the first evaluation, and were regularly followed up based on the database, with which a related study was published in 2020 [8]. To be enrolled, individuals had to be at least 14 years old and diagnosed with epilepsy according to the published criteria [13] at least one year before the enrollment.

Individuals were excluded if they (1) self-reported seizures within 24 h before completing the questionnaires at either time point (see Sect. Questionnaires); (2) failed to submit questionnaires at either of the time points; (3) had a history of mental retardation, alcohol or drug abuse, or uncontrolled psychosis; or (4) were unable to read or understand the questionnaires. They were also excluded if relevant clinical information was missing.

Epilepsy was classified as generalized, focal or unknown based on published criteria [14]. The patients were defined as drug-resistant if the participant had been treated with at least two appropriate anti-seizure medications [15]. Seizure freedom was defined as having no seizures for at least 12 months or a period equal to three times the longest pre-treatment inter-seizure interval [15].

### Questionnaires

Participants completed the following questionnaires online (via WeChat) twice: once during the period of December 1 to 15, 2022, which was before the outbreak (baseline), and the other during the period of January 15 to February 15, 2023, which was during the outbreak.

#### Clinicodemographic questionnaire

The sociodemographic data were collected before the outbreak. The participants completed a custom-designed questionnaire that collected items on age, sex, education level, and household income, medical history, clinical information on epilepsy. The clinical information on epilepsy included epilepsy classification, type of seizure, antiseizure medication, seizure frequency, the total number of seizure onsets during the outbreak and the last seizure onset.

At the second assessment during the outbreak, the participants completed a custom-designed questionnaire including seizure onset during the past 30 days, infection with SARS-CoV-2, diagnosis with COVID-19, neurological symptoms and mode of follow-up (online or offline).

The control group completed a custom-designed questionnaire collecting information of age, sex, education level and financial circumstances.

#### Kessler psychological distress scale

The Mandarin version of the 6-item Kessler Psychological Distress Scale (K-6) was used to assess nonspecific psychological distress during the past month, including symptoms of anxiety and depression. This Scale has been used to screen for psychological distress in healthy people [16, 17] and in individuals with epilepsy [8]. Each item had 5-point response options from 0 to 4, and the scores were summed to obtain a total score of 0 to 24; higher scores indicate greater psychological distress. Participants were classified into low psychological distress (0–7), mild distress (8–12), or severe distress (13–24) based on the total scores.

### Statistical analysis

Data were analyzed with the SPSS 25.0 (IBM, Chicago, IL) software. Categorical variables are reported as numbers and percentages; continuous variables are presented as means and standard deviations if normally distributed or as median (interquartile range) if skewed. Intergroup differences were assessed for significance using Student’s *t* test or the chi-squared test. Multivariate logistic regression was used to identify factors that were independently associated with exacerbation of psychological distress, defined as an increase in psychological distress severity (e.g. from “low” to “mild” or from “mild” to “severe”); or that were associated with severe psychological distress. The multivariate model contained variables with *P* < 0.1 in the univariate analysis. Risk factors were assessed in terms of odds ratio (OR) and 95% confidence interval (CI).

## Results

Of the 586 individuals who were treated at our epilepsy center during the recruitment period, 363 were excluded because of the lack of assessments before or during the outbreak (*n* = 238); ages younger than 14 years (*n* = 109); refusal to participate (*n* = 13); incomplete clinical data (*n* = 3). At last, 223 epilepsy patients (101 males, 45.3%) were included, with a mean age of 30.59 ± 10.95 years (range, 14–77 years) (Table 1). There were 218 healthy controls enrolled in the control group. The patients and controls showed no significant difference in age, marriage status, education level or mental illness. Patients reported significantly lower family incomes than the controls, which is consistent with our previous report [8].

**Table 1:** Clinicodemographic characteristics of the epilepsy group and the control group

Characteristics	Epilepsy group *n* = 223	Control group *n* = 218	*P* value
Male	101 (45.3)	94 (43.1)	0.701
Age, years	30.59 ± 10.95	32.06 ± 10.71	0.156
Married	100 (44.8)	79 (36.2)	0.081
Years of education			
≤ 12	98 (43.9)	80 (36.7)	0.145
> 12	125 (56.1)		
Monthly household income, RMB*			** < 0.001**
0–4999	154 (49.1)	58 (26.6)	
5000–9999	59 (26.5)	78 (35.8)	
10,000–19,999	8 (3.6)	35 (16.1)	
≥ 20,000	2 (0.9)	47 (21.6)	
History of mental illness	12 (5.4)	4 (1.8)	0.072
Epilepsy type			
Generalized	43 (19.3)		
Focal	169 (75.8)		
Unclassified	11 (4.9)		
Drug-resistant epilepsy	51 (22.9)		
Total number of antiseizure medications ever taken	2 (1,6)		
Tested positive for SARS-CoV-2 infection	127 (57.0)	174 (79.8)	** < 0.001**
Seizure onset within previous 30 days			
Before the outbreak	50 (22.4)		
Before the second questionnaire (during the outbreak)	66 (29.6)		
Kessler-6 score			
Before the outbreak	5 (5, 2)		
Before the second questionnaire	9 (24, 6)	2 (24, 0)	** < 0.001**

Values are *n* (%), mean (SD), or median (maximum, minimum)

### Neurological symptoms of epilepsy during the COVID-19 pandemic

**Fig. 1:**
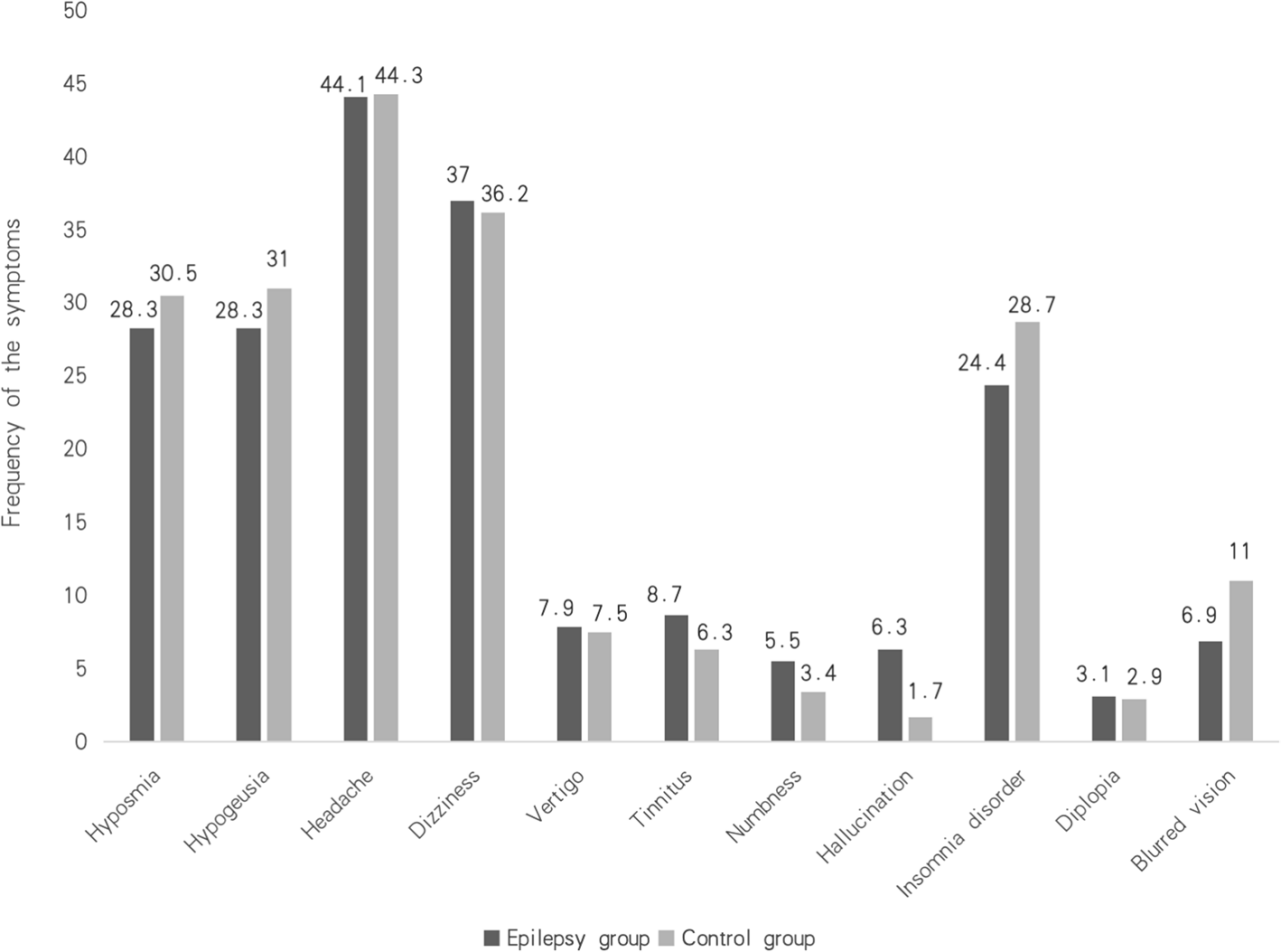
New-onset neurological symptoms among individuals with epilepsy who reported to be tested positive for SARS-CoV-2 during the outbreak (*n* = 127) compared with the control group (*n* = 174) during the Omicron outbreak. There was no significant difference in the frequency of the new-onset neurological symptoms between the two groups

A total of 187 respondents in the epilepsy group reported “common cold” symptoms, of whom 127 reported to be tested positive for SARS-CoV-2 infection during the outbreak. Both the epilepsy patients and the healthy controls reported various newly onset neurological symptoms during the outbreak (Fig. 1), with no significant difference between the two groups.

The frequency of individuals who suffered seizures within the previous 30 days was significantly greater during the outbreak than at the baseline period (*P* < 0.001), regardless of whether the individuals tested positive for SARS-CoV-2 during the study period (Table 1).

### Psychological distress

**Fig. 2:**
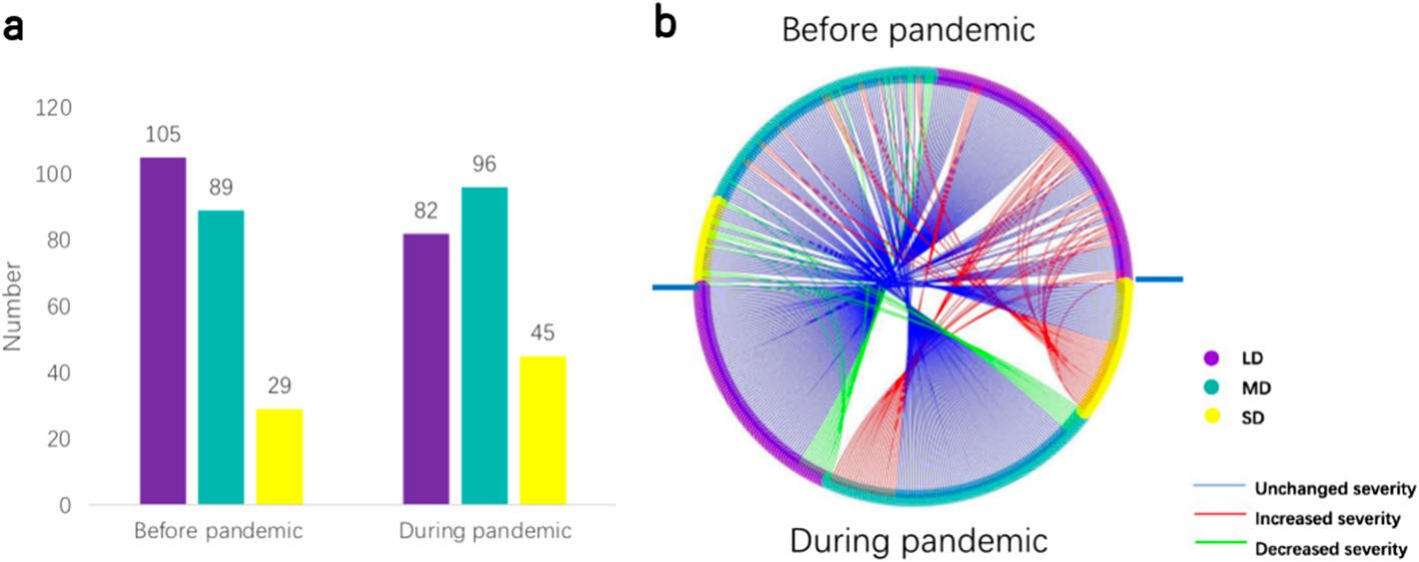
Distribution of participants (*n* = 223) by the severity of psychological distress before and during the Omicronoutbreak. **a** Numbers of participants categorized as having low, mild or severe distress. **b** Participant trajectories based on the severity of psychological distress, from before the outbreak (above the horizontal midline) to during the outbreak (below the midline). LD: Low distress, purple; MD: Mild distress, green; SD: Severe distress, yellow. Blue lines represent participants whose severity did not change (*n* = 156); red lines represent participants with increased severity (*n* = 48); green lines represent participants with decreased severity (*n* = 19)

In the epilepsy group, scores increased on all K-6 items during the outbreak, and the mean total score increased significantly from 8.52 ± 0.23 to 9.93 ± 3.98 (*P* < 0.001, Table 1 and Additional file 1: Table S1). In the healthy control group, the total score during the outbreak was 5.146 ± 0.35, which was significantly lower than that of the epilepsy group (*P* < 0.001, Table 1). The total K-6 score increased in 92 epilepsy patients, and the increase was sufficient to move 48 of them to the next higher category of psychological distress, such as from “low” to “mild” or from “mild” to “severe” (Fig. 2). The frequency of severe psychological distress, defined as a total K-6 score > 12, increased significantly during the outbreak (20.2% vs 13.0%,* P*= 0.042). In the control group, the frequency of severe psychological distress was 6.9% during the outbreak while it was 1.6% in the last three years before the outbreak [8].

The median total K-6 scores, either before or during the outbreak, did not differ significantly between individuals who tested positive for SARS-CoV-2 or not during the study period (Supplementary Table 1).

### Risk factors for exacerbation of psychological distress

Univariate analysis showed that monthly household income < 5000 RMB [18] (OR = 0.517, 95% CI 0.241–1.111, *P* = 0.091), use of valproate (OR = 0.408, 95% CI 0.202–0.826, *P* = 0.013) and seizure onset within 30 days before the second questionnaire (OR = 0.449, 95% CI 0.231–0.871, *P* = 0.018) were associated with aggravated psychological distress (Table 2). The significant association remained only for the last variable after multivariate logistic regression analysis (OR = 0.302, 95% CI 0.123–0.741, *P* = 0.009).

**Table 2:** Exploration of factors associated with an increase in the category of psychological distress severity during the Omicron outbreak^*^

Factor	Odds ratio (95% CI)	*P*
Monthly household income < 5000 RMB	0.517 (0.241, 1.111)	0.091
Use of valproate	0.408 (0.202, 0.826)	0.013
Seizure onset within 30 days before second questionnaire	0.449 (0.231, 0.871)	0.018

*CI* confidence interval

^*^Only factors associated with *P* < 0.1 are shown

### Risk factors associated with severe psychological distress during the outbreak

Univariate analysis revealed four factors associated with severe psychological distress during the outbreak (Table 3): monthly household income < 5000 RMB (OR = 0.415, 95% CI 0.182–0.946, *P* = 0.036), severe psychological distress before the outbreak (OR = 0.067, 95% CI 0.027–0.162, *P* < 0.001), seizure onset within 30 days before the second questionnaire (OR = 0.270, 95% CI 0.137–0.533, *P* < 0.001), and use of clonazepam (OR = 0.327, 95% CI 0.099–1.085, *P* = 0.068) or perampanel (OR = 0.238, 95% CI 0.090–0.628, *P* = 0.004). The first three variables remained significant after multivariate logistic regression analysis (Table 4).

**Table 3:** Exploration of factors associated with severe psychological distress during the Omicron outbreak^*^

Factor	Odds ratio (95% CI)	*P*
Monthly household income < 5000 RMB	0.415 (0.182, 0.946)	0.036
Use of clonazepam	0.327 (0.099, 1.085)	0.068
Use of perampanel	0.238 (0.090, 0.628)	0.004
Severe psychological distress before the outbreak	0.067 (0.027, 0.162)	< 0.001
Seizure onset within 30 days before second questionnaire (during outbreak)	0.270 (0.137, 0.533)	< 0.001

^*^Only factors associated with *P* < 0.1 are shown

**Table 4:** Multivariate logistic regression analysis of independent predictors of severe psychological distress during the outbreak

Predictor	OR	95% CI	*P*
Monthly household income < 5000 RMB	0.252	(0.064,0.998)	0.048
Severe psychological distress before the outbreak	0.067	(0.026, 0.174)	< 0.001
Seizure onset(s) within 30 days before the second questionnaire (during outbreak)	0.356	(0.157, 0.805)	0.013

Reference conditions for OR calculations: without hallucination or insomnia disorder; no perampanel use; no seizure onset within 30 days; no severe psychological distress

*Abbreviations*: *OR* odds ratio, *CI* confidence interval

## Discussion

To our knowledge, this is the first study on the change of psychological distress in Chinese individuals with epilepsy after the adjustment of COVID-19 prevention and control measures in December, 2022, which led to high rates of infection with SARS-CoV-2. Our results suggest that a national pandemic substantially increases the risk of severe psychological distress among individuals with epilepsy, especially those with household income below 5000 RMB or with uncontrolled seizures. Patients with Kessler scores > 12 should consult a psychiatrist, especially during major disasters such as a pandemic.

COVID-19 can affect many more systems in addition to the respiratory system, and more than 30% of patients present neurological symptoms [19–22]. The four most frequent neurological symptoms after SARS-CoV-2 infection in our participants were dizziness, headache, hyposmia, and hypogeusia, similar to the findings of studies on individuals with epilepsy [19] and on healthy individuals [19–22]. In fact, headaches, hyposmia, hypogeusia and fatigue are commonly reported by individuals suffering "long COVID” [23, 24]. These neurological symptoms may result from the passing of virus through the blood-brain barrier, or may be secondary effects from pathology in other organs or systemic inflammatory reactions [20, 25]. Whatever the cause, our study highlights the need to carefully examine individuals with epilepsy for potential symptoms of long COVID [24].

Although the pandemic appears to be associated with a significant increase in psychological distress regardless of SARS-CoV-2 infection or not, only 20.2% of our participants reported severe psychological distress during the Omicron outbreak, of whom 44.4% already showed severe distress before the outbreak. In contrast, a 2020 study of individuals with epilepsy around the pandemic reported that 57.1% had severe distress [25]. The smaller percentage in the present study may reflect that we never closed the epilepsy center during the pandemic, so patients were able to receive in-person or on-line counseling throughout the pandemic. Such continuity of care may provide an important outlet for relieving psychological distress [26]. The continuity of care is particularly needed in a global pandemic because of the prolonged impact and restrictions on social and professional life [26].

The seizure severity in our participants worsened during the outbreak, regardless of SARS-CoV-2 infection or not. Seizure worsening was also reported in a study of Italians with epilepsy [26, 27]. However, it is unclear to what extent such worsening is related to the neurotropism of SARS-CoV-2 or to the neurosymptomatology of COVID-19 [28]. The potential reason for the increased seizure severity may be the neurotropism of SARS-CoV-2, and seizures have been associated with COVID-19 [28]. Another potential reason may be the barrier to receiving care during the pandemic, and such disruption has been documented in many parts of the world [25, 27, 29].

In this study, seizure onset within 30 days before the second administration of questionnaire (during the outbreak) was an independent risk factor not only for severe psychological distress, but also for increased psychological distress severity. This is inconsistent with a previous analysis from our center, conducted entirely before the Omicron outbreak, which showed that seizure onset within the previous 30 days was unrelated to the psychological distress. The discrepancy may be due to the different study populations. In that study, only 1.2% of the individuals with epilepsy had previous contacts with someone who tested positive for SARS-CoV-2, and only one individual had been diagnosed with COVID-19. In the present study, more than 80% of individuals presented cold symptoms, and more than half of patients reported to be tested positive for SARS-CoV-2 [8]. The discrepancy may also reflect the observed increase in seizure frequency during the outbreak, which may be due in part to the restricted access to healthcare and support. These considerations imply that seizure control during a pandemic is important for ameliorating psychological distress.

Professional restrictions during the pandemic have affected the incomes of many households [30, 31], which may help explain why household income below < 5000 RMB in our sample was a significant risk factor for severe psychological distress. Financial problems have been linked to anxiety and depression among individuals with epilepsy [27], and household income has been reported as the most important risk factor for mental problems in the general population in many countries [32, 33].

Our findings should be interpreted with caution. Due to the sudden outbreak, it is impossible to foresee the recruitment of healthy controls. Therefore, we did not compare the epilepsy patients with healthy controls before the outbreak. In addition, we did not exclude the possibility that some participants in our study were infected with SARS-CoV-2 but did not know it. Similarly, we could not exclude that some participants who reported to be tested positive for the virus did not actually experience infection. In fact, all our data were self-reported in this study; therefore, more objective measures of psychological distress and neurological symptoms should be considered in future studies.

## Conclusions

Our analysis suggests that the COVID-19 pandemic has substantially increased psychological distress of individuals with epilepsy, regardless of the viral infection or not. Various neurological symptoms similar to those of “long COVID” appeared for the first time among these individuals during the Omicron outbreak, highlighting the need for clinicians to screen carefully for this condition. Management of epilepsy during the pandemic or a similar major disaster should focus on the control of seizures and maintenance of mental health, especially among those with monthly household incomes below 5000 RMB, suffering uncontrolled seizures and having a history of severe psychological distress.

## Supplementary Information

**Additional file 1: Supplementary Table 1.** Scores on the Kessler Psychological Distress Scale for individuals with epilepsy (*n* =223) before and during the Omicron outbreak.

## Data Availability

All data are available from the corresponding author upon reasonable request.

## Abbreviations

COVID-19: Corona Virus Disease 2019
PWE: People with epilepsy
SARS-CoV-2: Severe Acute Respiratory Syndrome Corona Virus 2
K-6: The 6-item Kessler Psychological Distress Scale

## References

[1] Fiest KM, Sauro KM, Wiebe S, Patten SB, Kwon CS, Dykeman J, et al. Prevalence and incidence of epilepsy: a systematic review and meta-analysis of international studies. Neurology. 2017;88:296–303. 27986877 10.1212/WNL.0000000000003509 PMC5272794

[2] Zhang Q, Li W, Li E, Yang X, Hao N, Yan B, et al. Disease awareness and dietary habits of patients with epilepsy in western China: a cross-sectional study. Acta Epileptologica. 2021;3(31).

[3] Forsgren L. Prevalence of epilepsy in adults in northern Sweden. Epilepsia. 1992;33:450–8. 1592018 10.1111/j.1528-1157.1992.tb01690.x

[4] Gaitatzis A, Sisodiya SM, Sander JW. The somatic comorbidity of epilepsy: a weighty but often unrecognized burden. Epilepsia. 2012;53:1282–93. 22691064 10.1111/j.1528-1167.2012.03528.x

[5] LaFrance WC Jr, Kanner AM, Hermann B. Psychiatric comorbidities in epilepsy. Int Rev Neurobiol. 2008;83:347–83. 18929092 10.1016/S0074-7742(08)00020-2

[6] Christensen J, Vestergaard M, Mortensen PB, Sidenius P, Agerbo E. Epilepsy and risk of suicide: a population-based case-control study. Lancet Neurol. 2007;6:693–8. 17611160 10.1016/S1474-4422(07)70175-8

[7] Layne Moore J, Elliott JO, Lu B, Klatte ET, Charyton C. Serious psychological distress among persons with epilepsy based on the 2005 California Health Interview Survey. Epilepsia. 2009;50:1077–84. 19260944 10.1111/j.1528-1167.2008.01996.x

[8] Hao X, Zhou D, Li Z, Zeng G, Hao N, Li E, et al. Severe psychological distress among patients with epilepsy during the COVID-19 outbreak in southwest China. Epilepsia. 2020;61:1166–73. 32353184 10.1111/epi.16544 PMC7267575

[9] Peron J. Direct and indirect impact of SARS-CoV-2 on the brain Human genetics. 2023:1–10. 10.1007/s00439-023-02549-x PMC10066989 37004544

[10] Del Brutto O, Wu S, Mera R, Costa A, Recalde B, Issa N. Cognitive decline among individuals with history of mild symptomatic SARS-CoV-2 infection: a longitudinal prospective study nested to a population cohort. Eur J Neurol. 2021;28:3245–53. 33576150 10.1111/ene.14775 PMC8014083

[11] Leung K, Lau EHY, Wong CKH, Leung GM, Wu JT. Estimating the transmission dynamics of SARS-CoV-2 Omicron BF.7 in Beijing after adjustment of the zero-COVID policy in November-December 2022. Nat Med. 2023;29(3):579–82. 36638825 10.1038/s41591-023-02212-y

[12] d’Orsi G, Mazzeo F, Ravida D, Di Claudio MT, Sabetta A, Lalla A, et al. The effect of quarantine due to Covid-19 pandemic on seizure frequency in 102 adult people with epilepsy from Apulia and Basilicata regions. Southern Italy Clin Neurol Neurosurg. 2021;203:106592. 33684674 10.1016/j.clineuro.2021.106592 PMC9759099

[13] Fisher RS, Acevedo C, Arzimanoglou A, Bogacz A, Cross JH, Elger CE, et al. ILAE official report: a practical clinical definition of epilepsy. Epilepsia. 2014;55:475–82. 24730690 10.1111/epi.12550

[14] Scheffer IE, Berkovic S, Capovilla G, Connolly MB, French J, Guilhoto L, et al. ILAE classification of the epilepsies: Position paper of the ILAE commission for classification and terminology. Epilepsia. 2017;58:512–21. 28276062 10.1111/epi.13709 PMC5386840

[15] Kwan P, Arzimanoglou A, Berg AT, Brodie MJ, Allen Hauser W, Mathern G, et al. Definition of drug resistant epilepsy: consensus proposal by the ad hoc task force of the ILAE commission on therapeutic strategies. Epilepsia. 2010;51:1069–77. 19889013 10.1111/j.1528-1167.2009.02397.x

[16] Kessler R, Andrews G, Colpe L, Hiripi E, Mroczek D, Normand S, et al. Short screening scales to monitor population prevalences and trends in non-specific psychological distress. Psychol Med. 2002;32:959–76. 12214795 10.1017/s0033291702006074

[17] Kessler RC, Green JG, Gruber MJ, Sampson NA, Bromet E, Cuitan M, et al. Screening for serious mental illness in the general population with the K6 screening scale: results from the WHO World Mental Health (WMH) survey initiative. Int J Methods Psychiatr Res. 2010;19(Suppl 1):4–22. 20527002 10.1002/mpr.310 PMC3659799

[18] Tang Q, Wang Y, Li J, Luo D, Hao X, Xu J. Effect of repeated home quarantine on anxiety, depression, and PTSD symptoms in a chinese population during the COVID-19 pandemic: a cross-sectional study. Front Psychiatry. 2022;13:830334. 35651827 10.3389/fpsyt.2022.830334 PMC9149163

[19] Asadi-Pooya AA, Emami A, Akbari A, Javanmardi F. COVID-19 presentations and outcome in patients with epilepsy. Acta Neurol Scand. 2021;143:624–8. 33590880 10.1111/ane.13404 PMC8014655

[20] Pantelis C, Jayaram M, Hannan A, Wesselingh R, Nithianantharajah J, Wannan C, et al. Neurological, neuropsychiatric and neurodevelopmental complications of COVID-19. Aust N Z J Psychiatry. 2021;55:750–62. 32998512 10.1177/0004867420961472 PMC8317235

[21] Roy D, Ghosh R, Dubey S, Dubey M, Benito-León J, Kanti Ray B. Neurological and Neuropsychiatric Impacts of COVID-19 Pandemic. Can J Neurol Sci. 2021;48:9–24. 32753076 10.1017/cjn.2020.173 PMC7533477

[22] Taquet M, Sillett R, Zhu L, Mendel J, Camplisson I, Dercon Q, et al. Neurological and psychiatric risk trajectories after SARS-CoV-2 infection: an analysis of 2-year retrospective cohort studies including 1 284 437 patients. Lancet Psychiatry. 2022;9:815–27. 10.1016/S2215-0366(22)00260-7 PMC9385200 35987197

[23] The Lancet Neurology. Long COVID: understanding the neurological effects. Lancet Neurology. 2021;20:247. 33743226 10.1016/S1474-4422(21)00059-4 PMC7969137

[24] Crook H, Raza S, Nowell J, Young M, Edison P. Long covid-mechanisms, risk factors, and management. BMJ (Clinical research ed). 2021;374:n1648. 34312178 10.1136/bmj.n1648

[25] Cross J, Kwon C, Asadi-Pooya A, Balagura G, Gómez-Iglesias P, Guekht A, et al. Epilepsy care during the COVID-19 pandemic. Epilepsia. 2021;62:2322–32. 34428314 10.1111/epi.17045 PMC8652685

[26] Kuroda N, Kubota T. Psychological impact of the COVID-19 pandemic for patients with epilepsy: a systematic review and meta-analysis. Epilepsy Behav. 2021;124:108340. 34600283 10.1016/j.yebeh.2021.108340 PMC9760102

[27] Assenza G, Lanzone J, Brigo F, Coppola A, Di Gennaro G, Di Lazzaro V, et al. Epilepsy Care in the Time of COVID-19 Pandemic in Italy: Risk Factors for Seizure Worsening. Front Neurol. 2020;11:737. 32719655 10.3389/fneur.2020.00737 PMC7350269

[28] Cabezudo-Garcia P, Ciano-Petersen NL, Mena-Vazquez N, Pons-Pons G, Castro-Sanchez MV, Serrano-Castro PJ. Incidence and case fatality rate of COVID-19 in patients with active epilepsy. Neurology. 2020;95:e1417–25. 32554773 10.1212/WNL.0000000000010033

[29] Guilhoto L, Mosini A, Susemihl M, Pinto L. COVID-19 and epilepsy: how are people with epilepsy in Brazil? Epilepsy Behav. 2021;122:108115. 34144461 10.1016/j.yebeh.2021.108115 PMC8412880

[30] Cao W, Fang Z, Hou G, Han M, Xu X, Dong J, et al. The psychological impact of the COVID-19 epidemic on college students in China. Psychiatry Res. 2020;287:112934. 32229390 10.1016/j.psychres.2020.112934 PMC7102633

[31] Van Hees S, Siewe Fodjo J, Wijtvliet V, Van den Bergh R, Villela E, da Silva C, et al. Access to healthcare and prevalence of anxiety and depression in persons with epilepsy during the COVID-19 pandemic: a multicountry online survey. Epilepsy Behav. 2020;112:107350. 32920373 10.1016/j.yebeh.2020.107350 PMC7481834

[32] Sturm R, Gresenz C. Relations of income inequality and family income to chronic medical conditions and mental health disorders: national survey. BMJ (Clinical research ed). 2002;324:20–3. 11777799 10.1136/bmj.324.7328.20 PMC61653

[33] Evans-Lacko S, Aguilar-Gaxiola S, Al-Hamzawi A, Alonso J, Benjet C, Bruffaerts R, et al. Socio-economic variations in the mental health treatment gap for people with anxiety, mood, and substance use disorders: results from the WHO World Mental Health (WMH) surveys. Psychol Med. 2018;48:1560–71. 29173244 10.1017/S0033291717003336 PMC6878971

