# Supplementary material for: Frequency and risk factors of psychological distress among individuals with epilepsy before and during the outbreak of the SARS-CoV-2 Omicron variant in China: an online questionnaire survey
Source: Acta Epileptol. 2024 Jan 15;6:2. doi: 10.1186/s42494-023-00146-z (PMC11960381; doi:10.1186/s42494-023-00146-z)
Supplement: Supplementary file 1 — Additional file 1: Supplementary Table 1. Scores on the Kessler Psychological Distress Scale for individuals with epilepsy (n =223) before and during the Omicron outbreak. [file 42494_2023_146_MOESM1_ESM.docx]

**Supplementary Table 1.** Scores on the Kessler Psychological Distress Scale for individuals with epilepsy (n =223) before and during the “omicron” outbreak

| **Item** | **Before** | **During** | ***P*** |
| --- | --- | --- | --- |
| Nervous | 1 (0,4) | 2 (0,4) | < 0.001 |
| Hopeless | 1 (0,4) | 1 (1,4) | 0.035 |
| Restless or fidgety | 2 (0,4) | 2 (0,4) | 0.007 |
| So depressed that nothing can cheer me up | 1 (0,4) | 1 (1,4) | 0.007 |
| Everything is an effort | 1 (0,4) | 1 (0,4) | 0.017 |
| Worthless | 1 (0,4) | 1 (0,4) | 0.010 |
| **Total score** | 8.52±0.23 | 9.93±3.98 | < 0.001 |
| **Severe psychological distress*, n (%)** | **29 (13.0%)** | **45 (20.2%)** | 0.042 |
| Test for SARS-CoV-2 infection  Positive  Negative | 8.22±3.52  8.91±3.52 | 9.65±3.68  10.29±4.34 | 0.151  0.237 |

Values are median (min, max) or mean **±** SD, unless otherwise noted.

* Defined as a total score more than 12.
